# Heart Team—the Indian perspective

**DOI:** 10.1007/s12055-018-0764-6

**Published:** 2018-11-16

**Authors:** Kunal Sarkar

**Affiliations:** Department of Cardiac Surgery, Medica Superspecialty Hospital, Kolkata, India

**Keywords:** Heart team, Multidisciplinary team, Coronary artery surgery

## Abstract

**Purpose:**

The European Society of Cardiology and the European Association for Cardio-Thoracic Surgery as well as the American College of Cardiology and the American Heart association have recognized the “Heart Team” as the best option for a patient centric treatment strategy and has granted a class I recommendation for its formation. The aim of this review is to discuss the evolution, scope and composition, the benefits, and problems inherent in its implementation in the Indian scenario.

**Methods:**

A review of articles on Heart Team from cardiac surgery as well as multidisciplinary meetings from other specialties was performed. Advantages of Heart Team formation and its implementation have been critically evaluated and its applicability to the Indian scenario considered in particular.

**Results:**

Heart Team formation is associated with many positives. Concern remains about the implementation of Heart Team approach in its true sense. Heart Team-led decisions are definitely patient centric despite multiple challenges in resource-limited environments.

**Conclusions:**

Despite the challenges, a multidisciplinary team approach in the form of Heart Team is recommended and its implementation possible in India. However, adjustments to the mechanism of implementation are required. Further research needs to focus on creating models for implementation and assessment of these models in terms of cost effectiveness, improved patient outcomes, and patient satisfaction in the process.

## Introduction

After an era of confrontation over percutaneous coronary intervention (PCI) and coronary artery bypass grafting (CABG), finally the cardiologists and the cardiac surgeons have started to collaborate to provide evidence-based patient centric care for coronary artery disease (CAD) in the form of Heart Team. This multidisciplinary collaborative approach to patient care provided by different stakeholders goes beyond guidelines and aims to provide a treatment option appropriate to the patient in question. The beneficiary of this approach is not just the patient, but also all the professionals involved, who in the process benefit from the exchange [1]. While the concept of Heart Team has many positives, its implementation is not without a multitude of problems, especially in India, where the work force providing this care is relatively small.

The aim of this review is to discuss the evolution, scope and composition, the benefits, and problems inherent in its implementation in the Indian scenario.

## Evolution of Heart Team

The concept of a Heart Team is gaining popularity among providers of therapies for CAD. Even though similar practices existed elsewhere, the concept of Heart Team was etched into cardiac surgery consciousness following the SYNergy between PCI With TAXUS and Cardiac Surgery (SYNTAX) trial where it was recognized that a team-based approach was required to optimize the management of complex care issues in patients presenting with CAD [2].

The reason why this patient centric Heart Team concept has evolved is a combination of patient care decisions becoming increasingly complex with development of different techniques to manage them, large volume of scientific data available from studies and multiple registries, increased awareness of patient choices, and a conscious effort to eliminate personal biases [1].

In the pre-angioplasty era, treatment for CAD was limited to medical therapy or CABG. The roles of the treating physicians and surgeons were clearly demarcated and the zone of conflict was virtually non-existent. It was with the advent of PCI and further scientific developments in the type of stents that led to an explosion in the number of studies comparing PCI with CABG and PCI with medical therapy alone. As the number of studies increased and the treatment options extended to medical therapy, PCI and CABG, the number of stakeholders providing the treatment also increased. From a cardiologist-cardiac surgeon model, there was the cardiologist, interventional cardiologist, and cardiac surgeon model that emerged where the latter two were vying to treat the same condition which was previously the domain of the cardiac surgeon alone. This brought about the question of appropriateness of the intervention and the superiority of one over the other. Moreover, invasiveness of CABG was brought into the focus for the first time by proponents of PCI. While most of the earlier studies clearly showed the superiority of CABG, the lesser invasiveness of PCI was propagated as a trade off, and that also brought in the largely ignored concept of patient choice in CAD. PCI was used not only to treat patients who were previously candidates for CABG but also encroached upon the domain of medical management. One example was in the setting of acute ST elevation myocardial infarction (STEMI). Previously, it was thought that the best treatment option for these patients was thrombolysis. However, now, PCI, where available and feasible and meeting the prescribed criteria, has become the preferred option [3]. Thus, not only the modalities of treatment had increased, but the specific situations in which they could or could not be implemented were being researched, with sometimes conflicting results. Task forces were set up to deal with the increasing complexity of the situation and guidelines were prepared based on the available evidence [4, 5]. It is, however, accepted that not all patients fall into a defined category and apart from medical complexities, financial, regional, and social influences formed the basis for individualizing each patient. This could only be done if all the experts and stakeholders sat together and discussed each individual situation. These factors finally culminated into the development of the Heart Team concept in the SYNTAX era in the field of cardiac surgery.

However, the concept of experts sitting together as a group to provide the optimal patient care has been in vogue much before and led to significant improvements in patient care in many other specialties, especially cancer services [6–8].

Even in cardiac surgery, in the field of transplantation [9], cardiac resynchronization [10], and congenital heart surgery [11], the concept of Heart Team has existed for quite some time prior to the SYNTAX era. Almost all major pediatric cardiac surgical centers regularly held multi-disciplinary team meetings, popularly called “Cath Conferences” for many years preceding the Heart Team concept in adult cardiac surgery. This again might be the result of complexity of the condition that required a consensus opinion of experts to provide the most suitable treatment. Thus, increasing complexities in treatment options and scenarios in coronary surgery above all may have dictated the inception of the Heart Team concept.

In essence, the Heart Team concept in cardiac surgery is the replication of the multidisciplinary team (MDT) concept used in the oncological and other settings. A team-based decision making in the form of a Heart Team was inevitable and a team-based decision making in the form of “Heart Team” provides the best option for a treatment strategy that is tailored to a particular patient. This has now been recognized by the European Society of Cardiology (ESC) and the European Association for Cardio-Thoracic Surgery (EACTS) as well as the American College of Cardiology (ACC) and the American Heart Association (AHA) by making the formation of Heart Team a class I indication [4, 5, 12, 13].

## Composition of the Heart Team

While it is recommended that the Heart Team for CAD consists of a cardiac surgeon, interventional cardiologist, and a general cardiologist, the composition of the Heart Team may vary. For instance, as in India, often, the patient’s general cardiologist and the interventional cardiologist are the same. Also, the composition varies with the clinical scenario. For instance, in transcatheter valve therapies (TVT), the team can include radiologists, neurologists, vascular physicians, and cardiac anesthesiologists [1].

## Scope of the Heart Team

The concept of Heart Team was initiated in coronary surgery but has now been extended to other areas of cardiac surgery as well. The guidelines mention that it is necessary that the multidisciplinary Heart Team discusses every patient and chooses the best individualized approach for management in many different valvular conditions like degenerated bio-prosthesis, transcatheter mitral and aortic valve treatment in patients at increased surgical risk, patients with moderate aortic regurgitation who undergo CABG or mitral valve surgery, and patients where aortic valve repair is being considered [14, 15].

Guidelines on valvular heart disease have stressed the importance of Heart Team in decision making for transcatheter aortic valve implantation (TAVI), as decision making for TAVI not only requires a risk/benefit analysis, but also requires taking into account factors like frailty or the presence of a porcelain aorta, which are not duly considered in the current risk stratification scores.

## Heart Team—does it improve outcomes?

The Heart Team perhaps allows the specialists to choose the most appropriate treatment modality for the patient, but the evidence that it leads to improvement in outcomes in patients with CAD is based on observational studies. This is reflected both in the ACC/AHA and the ESC/EACTS guidelines, where the class of recommendation is 1 but the level of evidence is C [13, 16]. However, there is a body of evidence showing definite improvement in clinical outcomes by implementation of a multidisciplinary approach from other cardiac areas, as well as from non-cardiac studies.

A meta-analysis looking at the impact of MDT meetings showed that up to 45% of patients discussed at MDT meetings experienced changes in diagnostic reports following the meeting. Patients discussed at MDT meetings were more likely to receive more accurate diagnosis and perhaps improved treatment [7]. MDTs have led to improvement in both process and clinical outcomes in a wide range of cancers like colorectal, head and neck, breast, esophageal, and lung cancers. Thus, MDTs have been associated with changes in clinical, diagnostic, and treatment decision-making and result in positive consequences for patients’ management in multiple dimensions [6].

An observational cohort pilot study carried out by researchers at the University of Pittsburgh Medical Centre examined the issues of safety and efficacy of implementing a multidisciplinary Heart Team approach for revascularization in patients with complex CAD. The authors compared the outcome of the pilot study with that of historical data and reported that outcomes of patients with complex CAD, undergoing the optimal treatment strategy recommended by the Heart Team, were similar to those of published national standards. Based on this, they concluded that implementation of the Heart Team approach for patients with complex CAD is safe and efficacious [17].

A prospective observational study that examined the role of multidisciplinary care approach versus conventional approach in managing patients requiring cardiac resynchronization therapy (CRT) revealed that the 2-year event-free survival in patients receiving CRT with the multidisciplinary approach was significantly higher [10].

Another study has shown that in-hospital mortality and 1-year mortality in patients admitted to the hospital for heart failure were significantly lower, if they were discussed by a Heart Team [18].

Considering that the Heart Team concept is relatively new to CAD, it may be a little while before studies confirming the benefit of integrated multidisciplinary approach emerge. For the time being, the level of evidence that the Heart Team actually leads to improvement in clinical outcomes is weak and there is no evidence, that it improves patient satisfaction, or indeed it is a more efficient system. The other area for where evidence needs to be gathered is whether the multidisciplinary approach is cost effective. This is perhaps even more relevant to the Indian setting, where there are major financial implications for the individual as well as institutions, in the light of various subsidized cardiac care schemes being launched by the government.

There is some suggestion that apart from allowing best possible treatment strategy for the patient, the Heart Team also might be useful as a medico-legal tool for the treatment providers. In the current scenario in India, where the scanner is firmly fixed on the medical profession in general and cardiac services in particular, Heart Team decisions are bound to have more impact on the credibility of the management strategy employed. It may not absolve the health care providers, but may provide them some protection by using a more scientific decision-making process in the interest of the patient, and in some ways, provide a shared responsibility. While no studies have been done to assess if Heart Teams reduce litigations, it appears intuitive that the Heart Team provides a sense of shared responsibility, rather than placing the entire onus of decision making on one individual. It may be reasonable to assume that shared decision making may reduce culpability for decisions gone wrong and subsequent medico-legal litigations [19].

There is no denying that apart from providing a way for cardiac surgeons and cardiologists to share the burden of decision making, such collaboration fosters an environment of teamwork, collegiality, and bidirectional exchange of knowledge that benefits all involved parties, but most importantly the patient [17].

## Problems with implementation

While the concept of Heart Team seems very appealing, the implementation is fraught with several issues to the extent, that in a recent review it was indeed questioned if the Heart Team concept could ever be a reality or was it a mere platonic illusion [20]. Institutions which have tried to implement the Heart Team approach have identified many different challenges in its implementation. One study reported that the time for the Heart Team meeting and the commitment from various specialists to participate in the meeting were major issues [17].

The issue of members of the Heart Team providing time and a commitment to the cause is perhaps the single most important barrier to implementation of the Heart Team concept in a resource poor country like India and merits critical evaluation. Compared to the western world, there is a definite smaller work force for a much larger population at risk. This applies to all the members which constitute the heart team.

In India, the overall physician density with respect to per 1000 population is 0.758, which is far behind United States of America (2.568), United Kingdom (2.825), Australia (3.496), Spain (3.872), Germany (4.191), and Greece (6.255) [21].

Cardiac service data show a similar trend. Available data suggests that India has a burden of 31.8 million patients, compared with 16.5 million patients in USA and 2.7 million in the UK. At the rate of 4% events per year in the total coronary population in India, 1.27 million acute coronary events occur per year as opposed to 0.63 million acute coronary events in the European union and 0.275 million heart attacks annually in UK [22]. In India, the age-adjusted cardiovascular mortality rates are 2–3 times greater than in the USA [23].

While the disease burden is high, the workforce is disproportionately smaller. At present, it is estimated that the number of cardiologists in India is 1 for every 300,000 persons [24], which is in sharp contrast to the USA, where the cardiologist-patient ratio is 48 per 100,000 [25].

Thus, a smaller work-force, confronted with greater demands, is overstretched. According to data provided by the national interventional council of India, there were 1200 cardiac catheterization labs in the country, of which 698 centers reporting their data carried out 373,579 coronary interventions in 2016. Forty-three percent of the Indian centers performed more than 1000 procedures, with 18% performing more than 2000 interventions in a year [26]. Using the definition provided for high volume centers (those performing more than 600 cases yearly) [27] and high volume operators [performed > 100 PCIs per year] [28], a vast majority of Indian centers and cardiologists would fall into the high volume category. In the United Kingdom, the guidelines suggest that primary PCI center must have two or more cardiac catheter laboratories, with a minimum of six Interventional Cardiologists preferably. [10] This further highlights the resource poor situation in India, where such requirements would be nothing short of an unaffordable luxury.

The same picture emerges for cardiac surgeons. Extrapolation of available data on the number of cardiac surgeons [29] and the population at risk [22] suggests that the ratio of cardiac surgeons and patient population in India is perhaps less than half of that in the USA. In reality, the work force might be even smaller.

With such a level of resource poor situation, the members making time for the Heart Team meetings in India are certainly the biggest challenge, and whether these limited resources be at all diverted from actual clinical care to Heart Team meetings may even be open to question.

It is not just the sheer volume of work that is a deterrent for Heart Team implementation. Majority of health care in India is privately funded and a delay in terms of length of stay translates into a higher expense for the patient. So, patient expectations for a Heart Team-led decision making have to be balanced in the Indian context by the expectation of a shorter hospital stay and lesser expense. Moreover, some of the current government schemes currently are heavily subsidized and may not make financial sense for the hospitals to carry out any revascularization procedures, let alone have a Heart Team meeting to discuss the appropriateness of the procedure.

One can even argue that even in the absence of a Heart Team, contrary to popular apprehensions, decision making is satisfactory. A study examining appropriateness of government funded coronary revascularization studied 600 patients from 28 hospitals in Karnataka, who obtained CABG or PCI. A total of 86.7% of these procedures were deemed appropriate, 3.65% inappropriate, and 9.63% were uncertain. The results exceeded the level of appropriate usage in the USA [30].

In existing models, while there is no provision for members of the Heart Team being incentivized, however they are still liable for their professional opinion, even though they may not have had any personal contact with the patient. Input to a treatment recommendation, even within a multidisciplinary meeting, with no face to face consultation, still constitutes a formal relationship and, as such, the contributing doctor is liable for the advice provided [31, 32]. So, this appears to be an extreme example of disincentivizing Heart Team participation. A recent review on the subject quite rightly pointed out that physical time spent on procedures continues to be reimbursed much more generously than the cognitive efforts put therein. Therefore, the question of incentivizing all the members of the team for their efforts cannot be ignored [20].

Engaging different physicians into a complex decision-making process and integrating and summarizing input from multiple viewpoints and finally accurately communicating the decisions and discussion to the patients and their families is also a major challenge [33].

Another impediment to Heart Team implementation in India is presence of standalone cath-labs, with no on-site surgical back up. A regular forum like a Heart Team in a non-surgical cardiology center will require a surgeon to travel to that unit, which impacts on the surgeon’s time for clinical care provision, and also has the disadvantage for the cardiologists that the surgical back up may at best be periodic and can cause significant time delays in providing care.

## Model for provision of Heart Team services

So, how can Heart Teams be implemented that allows adequate utilization in a resource poor environment? The answer needs pondering over some difficult issues and making many adjustments and perhaps considering many different models tailored to specific institutions. It is unlikely that in a vast country like India, with multiple tiers of care and mode of reimbursements, any one single model can be implemented successfully.

The first question one has to consider is how likely is it that in India every patient requiring cardiac surgery is offered a Heart Team consultation. Secondly, given our resource poor status, do all patients undergoing coronary angiogram need a Heart Team consultation? Or should it be used in a well-defined set of conditions agreed upon and incorporated in the standard operating protocol of the Heart Team. Should Heart Team discussions be the standard of care, irrespective of reimbursements or should it be offered to patients as an add on and billed for?

Before the Heart Team delivery model is considered, it is perhaps prudent to examine the logistics involved. Limited data on the logistics reveal significant institutional variation. The frequency at which these meetings are held varies widely. While there are centers which have a Heart Team meeting every day [34], there are others where it takes place twice a week [35]. The number of surgeons and cardiologists present in these meetings are also quite variable, with average number of physicians (both cardiologists and cardiac surgeons) present being reported as 4 in one study [34], while in another, a median of 2 cardiac surgeons and 4 cardiologists were present at each meeting [35].

In India, the model of care is such that the volume of referral of patients is often skewed significantly to particular surgeons and interventional cardiologists. While it would be quite reasonable to expect that the surgeons and interventional cardiologists, ultimately responsible for delivering the intervention, should attend these Heart Team meetings, realistically it is hard to imagine how a surgeon performing three to four, sometimes even 5 cases every day is going to be able to attend these meetings and how much time can they be expected to provide on a daily basis, coinciding with an equally busy cardiologist. If these meetings were to be attended by other team members, would the decisions made in the meeting be reproducible and acceptable to the surgeons or the interventional cardiologists? Most importantly, would the patient feel comfortable and reassured that the decision making and the care providing teams are different?

There is very limited data on what constitutes an optimum allocated time for discussion of each patient. One of the centers reported reviewing an average of 9 patients every session, which was for 2 h. This makes the time allocation for each case discussion around 15 min. A high-volume institution in India would be performing an average of 25 angiograms every day. Given an average time of 15–20 min for each case discussion, the Heart Team meeting could require up to 8 h, almost one working day to discuss the angiograms churned out of a day’s work. In a country, where there is a dearth of cardiac surgeons as well as cardiologists, this does not appear to be proper utilization of resources. Even in smaller centers, time allocation for Heart Team meeting is an issue because the number of personnel is far less than a high-volume center. Thus, appropriate resource utilization assumes far greater importance in a resource-deficient environment like India. How often does the Heart Team meet, how much time is allocated to each discussion, and how many patients are discussed in one session, who attends these sessions, need to be determined on an individual basis. Every center has to work out its own model.

## Heart Team models in coronary disease

Basically, there are two potential models. The first model is all inclusive heart care model, where every angiogram is discussed by the Heart Team. The second model is selective provision of Heart Team expertise.

“Heart team care for all” model may be idealistic but may not be the most practical, especially in the context of India. Apart from being impractical, this approach in some circumstances, due to the compulsion to pass every angiogram through a Heart Team, might even delay patient care. However, this model has its merit and could potentially be the preferred approach in low volume centers. The heart team members may not be under pressure to perform large number of procedures and can focus on providing “heart team for all” philosophy. Days could be decided when Heart Team meetings would be carried out with provision for scheduling urgent meets.

However, for larger volume centers, a more practical model would be to select the cases that merit a Heart Team discussion. The obvious question then would be what should be the criteria for provision for this care?

The Heart Team should be writing an institution-specific guideline as to which cases can be referred for Heart Team meetings. The guideline has to be center specific, e.g., some centers choose to discuss patients with unprotected left main CAD, 3-vessel CAD, 2-vessel CAD involving the proximal left anterior descending (LAD) artery, proximal LAD disease in patients with diabetes mellitus, and any complex CAD which could be reasonably approached by either a percutaneous or surgical strategy [34]. The ESC/EACTS guidelines for myocardial revascularization recommendations for cases that should undergo Heart Team discussion include presence of left main stem (LMS) disease, proximal segment of its LAD branch, and anatomical patterns of disease in which CABG may confer prognostic advantage [12].

A selective model for Heart Team discussion would, however, require these centers to perhaps consider the option of ad hoc PCI. Ad hoc PCI is defined as a therapeutic intervention performed within the same procedure as the diagnostic coronary angiography [36]. Ad hoc PCI though convenient, cost-effective, and safe has the potential to be misused and it has been shown that many patients undergoing ad hoc PCI were potential candidates for CABG [37]. The Heart Team has to lay down strict protocols for considering ad hoc PCI in stable patients, in accordance with current guidelines defining specific anatomical criteria and clinical subsets that may be—or should not be—treated ad hoc. The indications for follow on PCI has to be formulated by the Heart Team as a standard operating protocol and may include emergency situations like addressing the culprit lesion in patients presenting with acute STEMI or elective cases with single- or double-vessel disease in line with the guidelines [38]. Complex pathologies in stable patients, including lesions of the LM or proximal LAD and three-vessel disease, should in general not be treated ad hoc, but discussed by the Heart Team.

It would certainly be discriminatory to exclude government subsidized scheme patients to be excluded from Heart Team discussion, but perhaps an add on charge could be negotiated with the insurance companies and government bodies. Remuneration for participants should be considered to ensure active participation.

## Heart Team models in valvular heart disease

There are currently three proposed models in the area of aortic valve replacement (AVR). The first model is where every patient, after the diagnosis of severe aortic stenosis (AS), is referred to the Heart Team. The Heart Team then considers the options of optimal medical therapy (OMT), transcatheter AVR (TAVR), and surgical AVR (SAVR). The second model involves a patient with AS, after being seen by a cardiologist, be referred directly to a cardiac surgeon, interventional cardiologist, or to the Heart Team. The third model is the model of a Heart Valve Center, where a referred patient is seen by both the cardiologist and the cardiac surgeon, who can then decide if the patient needs further discussion in a Heart Team. [39]

Considering that most centers in India currently do not have a TAVR program, none of the above models may be applicable for the time being. However, if there is a debate between OMT and SAVR, the services of the Heart Team should be sought. As TAVR becomes more popular, the centers can choose one of the three models or even come with a model that better serves the Indian patients.

## Proposed heart care model for standalone catheterization suites

This situation is not unique to India, as such units are seen even in other countries, like the United Kingdom. The options include either a close liaison with a cardiac surgical unit and surgeons may visit the stand alone unit on fixed days. The other option would be to develop teleconferencing facilities. These facilities allow for a virtual MDT and can incorporate several members of the surgical center team, as well as by high volume PCI operators from other units, who can make additional contributions to any case discussion.

## Summary

Heart Team approach to patient care has many positives. Concerns remain about the implementation of Heart Team approach in its true sense. Heart Team-led decisions are definitely patient centric and despite multiple challenges in resource-limited environments, a multidisciplinary team approach can be incorporated into clinical practice in India and other developing nations. However, this needs making some adjustments to the mechanism of implementation, while maintaining the sanctity and spirit of the process. Further research needs to focus on creating models for implementation and assessment of these models in terms of cost effectiveness, improved patient outcomes, and patient satisfaction in the process.
